# Sleep enhances gamma oscillations in the seizure onset zone and broadband activity in the irritative zone of focal cortical dysplasia

**DOI:** 10.1002/epi4.70215

**Published:** 2026-01-20

**Authors:** Mohammad F. Khazali, Nina Schwoon, Armin Brandt, Matthias Dümpelmann, Yiwen Li Hegner, Nicolas Roehri, Dirk‐Matthias Altenmüller, Victoria San Antonio‐Arce, Peter C. Reinacher, Julia M. Nakagawa, Soroush Doostkam, Theo Demerath, Horst Urbach, Andreas Schulze‐Bonhage, Marcel Heers

**Affiliations:** ^1^ Epilepsy Center, Medical Center – University of Freiburg, Member of the European Reference Network for Rare and Complex Epilepsies EpiCARE, Faculty of Medicine University of Freiburg Freiburg im Breisgau Germany; ^2^ BrainLinks‐BrainTools Center University of Freiburg Freiburg Germany; ^3^ Department of Microsystems Engineering—IMTEK University of Freiburg Freiburg Germany; ^4^ Department of Neurology and Epileptology, Hertie Institute for Clinical Brain Research University of Tübingen Tübingen Germany; ^5^ EEG and Epilepsy Unit, University Hospitals and Faculty of Medicine University of Geneva Geneva Switzerland; ^6^ Department of Stereotactic and Functional Neurosurgery, Medical Center – University of Freiburg, Faculty of Medicine University of Freiburg Freiburg im Breisgau Germany; ^7^ Fraunhofer Institute for Laser Technology Aachen Germany; ^8^ Department of Neurosurgery, Medical Center – University of Freiburg, Faculty of Medicine University of Freiburg Freiburg im Breisgau Germany; ^9^ Translational Epilepsy Research, Department of Neurosurgery, Faculty of Medicine University of Freiburg Freiburg im Breisgau Germany; ^10^ Institute of Neuropathology, Medical Center – University of Freiburg, Faculty of Medicine University of Freiburg Freiburg im Breisgau Germany; ^11^ Department of Neuroradiology, Medical Center – University of Freiburg, Faculty of Medicine University of Freiburg Freiburg im Breisgau Germany

**Keywords:** fast oscillations, focal cortical dysplasia, focal epilepsy, intracranial EEG, sleep

## Abstract

**Objective:**

Focal cortical dysplasia (FCD) is a leading cause of drug‐resistant epilepsy and is associated with sleep‐related seizures, yet the underlying electrophysiological mechanisms during different brain states remain poorly understood. We investigated whether fast oscillations (FOs) within the seizure onset zone (SOZ) and irritative zone (IZ) change significantly during sleep compared with wake in FCD patients. We analyzed multiple frequency bands—beta (14–20 Hz), gamma (40–80 Hz), ripple (80–250 Hz), and broadband BGR (14–250 Hz) to provide comprehensive information about sleep‐related changes. We hypothesized that sleep‐related FO changes would be associated with sleep‐related epilepsy, frontal location, or FCD type II.

**Methods:**

We examined intracranial EEG (iEEG) recordings from 22 FCD patients undergoing presurgical evaluation between 2010 and 2023, with a mean age of 25.3 ± 12.8 years and a disease duration of 17.7 ± 12.3 years. Using semiautomated detection, we compared FO rates between wake and sleep epochs, focusing on contacts within IZ and SOZ. Distance‐based multivariate analysis of variance (MANOVA) was employed for patient‐level analysis, accounting for spatial organization and enabling multiband evaluation.

**Results:**

Analysis of 67 ± 28 bipolar iEEG contact pairs per patient revealed distinct sleep‐related patterns. In SOZ, gamma oscillations showed significant increases in 10/22 patients (45%), followed by BGR in 7/22 patients (32%). IZ exhibited stronger changes, with BGR and gamma showing significance in 13/22 patients each (59%) with high concordance. Gamma oscillation rates in SOZ increased in patients with confirmed sleep‐related epilepsy (*p* < 0.05), while no associations were found with frontal location or FCD type II.

**Significance:**

Gamma oscillations showed robust sleep‐related increases in SOZ, while gamma and BGR frequencies demonstrated strong changes in IZ, often occurring simultaneously. These findings suggest gamma oscillations, complemented by BGR analysis, may qualify as markers for characterizing sleep‐related changes in FCD patients, potentially advancing understanding of mechanisms underlying sleep‐related seizures.

**Plain Language Summary:**

Focal cortical dysplasia (FCD) is a brain malformation that causes difficult‐to‐treat epilepsy, with patients experiencing seizures mainly during sleep. We studied electrical brain waves in 22 FCD patients using electrodes placed directly within or on the brain during pre‐surgery evaluation. We compared brain wave activity between wake and sleep, focusing on fast brain waves. We found that fast brain waves, especially gamma waves, increased significantly during sleep in brain areas where seizures start, particularly in patients whose seizures occur mainly during sleep. These findings potentially help us better understand why seizures happen more often during sleep in FCD patients.

**Key points**

1. Intracranial EEG reveals gamma oscillations in seizure onset zones increase significantly during sleep in focal cortical dysplasia.
2. Gamma increases during sleep were strongest in patients with sleep‐related epilepsy, linking oscillations to clinical seizure patterns.
3. Broadband activity strengthens in irritative zones during sleep, providing insights into sleep‐modulated epileptic networks.
4. Gamma and broadband analysis may serve as biomarkers for characterizing sleep effects in presurgical epilepsy evaluation.

## 1 INTRODUCTION

### 1.1 Presurgical epilepsy diagnostics

Epilepsy is one of the most common chronic neurological diseases.^1^ Approximately 30% of patients with focal epilepsy suffer from drug resistance,^2^ defined as the absence of sustained seizure freedom after adequate therapy with at least two antiseizure medications (ASMs).^3^ Epilepsy surgery can be a viable therapeutic option in these patients, achieving seizure freedom in 60%–65% of patients after 2 years, particularly for patients with lesions visible in the MRI.^4^ Pre‐surgical diagnostics determine the characteristics of the epileptic focus and the feasibility of epilepsy surgery. It includes high‐resolution MRI of the brain, neuropsychological testing, and noninvasive video‐EEG monitoring to identify the irritative zone (IZ) where interictal epileptiform discharges occur and the seizure onset zone (SOZ), where seizures arise.^5^ In a subset of patients, invasive video‐EEG monitoring with intracranial EEG (iEEG) needs to be added to delineate the SOZ and the IZ at a higher spatial resolution.^5^ Among the pathologies requiring such detailed pre‐surgical evaluation, focal cortical dysplasia (FCD) represents one of the most frequent causes of drug‐resistant epilepsy.

### 1.2 Focal cortical dysplasia and sleep‐related epilepsy

Understanding the pathophysiology of FCD and its relationship to sleep is crucial for improving therapeutic strategies in drug‐resistant epilepsy.^6^, ^7^, ^8^ Five to twenty‐five percent of patients with focal epilepsy suffer from FCD.^9^, ^10^ FCDs are cortical developmental disorders caused by a malfunction in neuronal migration and differentiation.^11^ Notably, FCD distinctly increases the risk of sleep‐related epilepsy (SRE),^8^ where the majority of seizures occur during sleep.^6^ Among the three subtypes of focal cortical dysplasia (FCD), type II is distinguished by the presence of dysmorphic neurons and, in the case of type IIB, balloon cells.^11^, ^12^

### 1.3 Differences of fast oscillations (FOs) between wake and sleep

The relationship between FCD and sleep‐related seizures is observed in intracranial EEG (iEEG) recordings by the presence of characteristic interictal patterns that occur between seizures. These patterns span a broad range of frequencies from 14 to 250 Hz^13^, ^14^ and differ markedly between wakefulness and sleep.^7^ Visual analysis reveals that repetitive sharp waves predominate during wakefulness, whereas rhythmic patterns in different frequency bands are more prevalent during sleep.^15^, ^16^ Semiautomatic detection methods can identify these interictal rhythmic patterns, known as FOs, in specific frequency bands: beta (14–40 Hz), gamma (40–80 Hz), and ripple (80–250 Hz).^17^, ^18^

Different frequency bands reflect distinct yet potentially overlapping aspects of FCD pathophysiology. While these bands have been individually studied in relation to the epileptic focus,^14^, ^19^, ^20^ their complex interplay during wake and sleep states remains poorly understood.

Sleep substantially increases seizure likelihood in many FCD patients,^21^ suggesting that vigilance‐state transitions promote pro‐ictal changes in neural physiology. FOs above 80 Hz spatially correlate with the epileptic focus and reflect disease severity in FCD patients,^22^ and show increased expression during sleep compared with wakefulness.^21^ We propose that sleep‐related increases in FO activity may serve as biomarkers of heightened seizure susceptibility. Previous study has identified patient‐specific dominant frequency bands within epileptogenic tissue,^14^, ^19^, ^20^ yet it remains unclear how vigilance states systematically modulate these frequency‐specific signatures and whether such modulation varies between the SOZ and the IZ. Characterizing broadband FO dynamics (beta, gamma, and ripple oscillations) across wake and sleep states may reveal whether vigilance‐dependent changes manifest as frequency‐specific biomarkers of heightened seizure susceptibility in FCD.

### 1.4 Hypothesis

We hypothesized that FO rates across a broad frequency range (14–250 Hz) within the SOZ and the IZ would show systematic differences between non‐rapid eye movements (NREM) sleep stages 2–3 and wakefulness. Therefore, a holistic analysis of the spatial correlations of broadband frequencies considering beta, gamma, and ripple oscillations would be more informative about sleep‐related changes of the epileptic focus consisting of the IZ and the SOZ than focusing on narrow bands such as beta, gamma, or ripple frequencies in isolation. In addition, we hypothesized that significant FO rate changes would be associated with sleep‐related epilepsy, frontal lobe epilepsy, and FCD type II histopathology.

## 2 PATIENTS AND METHODS

### 2.1 Patient selection

All 22 patients underwent iEEG at the Epilepsy Center of the University Hospital Freiburg between 2009 and 2023. Initially, 31 patients above the age of 12 years with either diagnosis of neocortical FCD based on MRI criteria or histopathological diagnosis of FCD were reviewed. The study was approved by the institutional ethics board of the University of Freiburg and limited to adolescents and adults above the age of 11 years, according to the ethics board approval. Patients gave written informed consent that their clinical data can be used for research projects. We confirm that we have read the Journal's position on issues involved in ethical publication and affirm that this report is consistent with those guidelines. Seven patients were excluded due to a lack of continuous iEEG data, and two patients were excluded because seizures were too frequent to select a sufficient interictal time interval for analysis. The mean age of patients was 25.3 ± 12.8 (mean ± standard deviation) years with a mean age at first manifestation of 11.6 ± 10.5 years and a disease duration of 17.7 ± 12.3 years. Twelve patients were female (more details on included patients in Table 1).

**TABLE 1:** Clinical characteristics of included patients.

ID	Electrode type	MRI visible lesion	Histopathology	Outcome	Resection complete	Seizure types
1	sEEG (6)	F, L (+)	N/A	1b (12)	Yes	FA, fbtc
2	sEEG (9)	F, L (+)	FCD IIB	1a (18)	Yes	FA, FIA, fbtc
3+	sEEG (6)	FB, R (+)	mMCD	1a (18)	Yes	FIA, fbtc
4	sEEG (7)	P, L (+)	FCD IIB	2a (14)	Yes	FA, fbtc
5	sEEG (8)	F, R (+)	FCD IIA	1a (72)	Yes	FA
6	sEEG (10)	OTB, R (+)	FCD IIA	1b (55)	Yes	FIA, fbtc
7+	sEEG (9)	F, R (+)	N/A	1a (45)	Yes	FIA
8+	G (8×6)	FO, L (+)	FCD IIB	1a (12)	Yes	FA, fbct
9+	sEEG (10)	F, R (−)	FCD IB	4b (56)	Yes	FA, fbct
10	G (8×8), strips (6)	F, L (+)	FCD IIA	1a (96)	Yes	FA, fbtc
11+	sEEG (13)	FT, R (+)	N/A	4b (12)^a^	No	FIA, fbtc
12+	sEEG (13)	OT, L (+)	FCD IB	1a (117)	Yes	FIA, fbtc
13+	G (8×8)	F, L (+)	N/A	1b (12)	No	FIA
14+	sEEG (13)	F, R (−)	FCD IB	4b (48)	No	FA, FIA, fbct
15+	G (8×8), strips (7)	F, R (−)	FCD IB	4b (12)	No	FA, FIA, fbtc
16+	G (8×8), strips (4)	OT, R (−)	FCD IIA	1a (72)	Yes	FA, FIA, fbtc
17+	sEEG (10)	C, R (+)	FCD IIB	4b (12)^b^	No	FA, fbtc
18	sEEG (11)	T, R (+)	FCD IIB	1a (12)	No	FIA
19	G (8×4), strips (8), sEEG (1)	O, L (−)	FCD IIA	2a (12)	No	fbtc
20	G (4×6), sEEG (1)	F, R (+)	FCD IIB	1b (24)	No	FA
21+	G (8×8), strips (1)	TP, L (−)	FCD IB	1a (24)	No	FA
22+	G (8×8), strips (6)	OT, R (−)	FCD IIA	1d (19)	Yes	FIA, fbtc

*Note*: Patient data and clinical findings, MRI visibility of the lesion, type of intracranial EEG electrode, localization of the lesion, histopathological findings, postsurgical outcomes according to the Engel classification and follow‐up time (in months), seizures after surgery, complete resection, seizure type. Outcome (follow‐up in months), electrode type (number of electrodes).

Abbreviations: (+) behind MRI‐positive lesion, lesion visible on MRI; +behind patient ID, sleep‐related epilepsy; C, cingulate gyrus; F, frontal; FA, focal aware; FB, fronto‐basal; fbtc, focal to bilateral tonic–clonic; FCD, focal cortical dysplasia; FIA, focal impaired awareness; FO, fronto‐opercular; FT, fronto‐temporal; G, Grid electrodes; L, left; N/A, not available; O, occipital; OT, occipito‐temporal; OTB, occipito‐temporo‐basal; P, parietal; R, right; sEEG, stereo‐EEG; sup. T, superior temporal gyrus; TP, temporo‐parietal.

^a^ 3a (3).

^b^ 3a (12) after second surgery, 1b (20) after third surgery.

### 2.2 Details of iEEG, histopathology, and postsurgical outcome

IEEG was recorded using either subdural grid/strip electrodes or intracerebral depth EEG electrodes. Subdural EEG electrodes had a contact diameter of 4 mm with an exposed contact surface diameter of 2.3 mm and a center‐to‐center inter‐contact distance of 10 mm (AD‐tech, Racine, WI, USA). Intracerebral depth electrodes had a contact length of 2 mm with a center‐to‐center inter‐contact distance of 4.5 mm and a contact diameter of 0.87 mm (AD‐tech, Racine, WI, USA). In five patients, DIXI electrodes were used with a contact length of 2 mm, thickness of <0.8, and 3.5 mm distance between contacts (Dixi‐medical, Marchaux – Chaudefontaine, France).

Thirteen patients underwent stereo‐EEG with multiple intracerebral depth electrodes, seven patients underwent subdural EEG, and two patients had a combination of both subdural and intracerebral depth. In 11 out of 22 patients, scalp EEG was recorded simultaneously with iEEG.

After iEEG, all patients underwent epilepsy surgery. Histopathological specimens were classified according to the International League Against Epilepsy (ILAE) classification.^11^ Histopathological examination revealed FCD type II in 12 patients, FCD type I in five patients, and mild malformation of cortical development (mMCD) in one patient. Please be aware that FCD type I is typically spatially extended with extended IZ and SOZ that often involve many iEEG contacts. Four patients were categorized based on MRI criteria by an experienced neuroradiologist (H.U.) because the neuropathological samples were insufficient for accurate histopathological classification.^23^

Favorable Engel class 1 postsurgical outcome was achieved in 15 of 22 (68%) patients. An unfavorable outcome occurred in 7 patients (Engel 2a: two patients, Engel 4b: five patients)^24^ at least follow‐up with a minimum follow‐up of 12 months (Table 1).

In accordance with the definition of sleep‐related epilepsy, where the majority of seizures occur during sleep, we established the criterion that two‐thirds of the seizures should occur during sleep during presurgical video‐EEG monitoring.^6^ Taking the video‐EEG monitoring as ground truth for sleep‐related epilepsy was chosen as reliable long‐term outpatient data on the number of seizures during sleep compared with wake are usually not available. In this regard, patient reports are typically inaccurate, as patients often do not remember nocturnal seizures. Based on the definition mentioned earlier, 13 patients were identified as having sleep‐related epilepsy.

### 2.3 MRI analysis and identification of iEEG electrode positions

In our project, four high‐resolution MRI sequences with isotropic voxels (edge length: 1 mm) were processed per patient. Three presurgical 3T MRI sequences (Trio or Prisma, Siemens, Erlangen, Germany) included a 3D T1 magnetization prepared (MPRAGE) sequence, a 3D fluid‐attenuated inversion recovery (FLAIR) sequence, and, when available, a magnetization prepared rapid gradient echo (MP2RAGE) sequence with MRI morphometry^25^ as part of the in house presurgical epilepsy MRI protocol.^26^ Additionally, all patients underwent a post‐implantation 1.5T MRI (Avanto, Siemens, Erlangen, Germany) after iEEG electrode implantation.

All four MRI sequences were processed in the Matlab Toolbox (Matlab 2018b, Mathworks, Natick, MA, USA) Brainstorm.^27^ In Brainstorm, the positions of three landmarks (left and right preauricular points and nasion) were estimated using a 12‐parameter affine transformation in SPM12^28^ aligned to the Montreal Neurological Institute ICBM 152 brain^29^ without spatial normalization of individual MRIs. After rigid co‐registration using SPM12, individual positions of iEEG electrode contacts were marked based on electrode positions, orientation, contact length, and inter‐contact distance. The cortical surface was tessellated and assigned to the Automatic Anatomical Labeling atlas^30^ using the Matlab toolbox CAT12^31^ as implemented in Brainstorm software. The FCD could be precisely visually identified in 15 patients.^23^

### 2.4 iEEG acquisition and analysis

For each patient, one interval during sleep and one interval during wakefulness were selected. These intervals each had a duration of 1 h, ended at least 15 min before and started at least 30 min after focal aware or focal impaired awareness seizures. After focal to bilateral tonic–clonic seizures, a minimum time interval of 2 h was required before the selected interval.

For sleep analysis, we aimed to select intervals predominantly consisting of NREM sleep of stages 2 or 3 according to the criteria of the American Society of Sleep Medicine and Rechtschaffen and Kales.^32^ Sleep–wake state differentiation was performed to compare fast oscillations across behavioral states. For patients with scalp EEG data (*n* = 11), the Persyst 14c wake–sleep feature (FDA‐cleared in version P15; validated with >94% accuracy for wake–sleep differentiation, Information for user Persyst 15) was used to classify sleep and wake intervals. For patients without scalp EEG (*n* = 11), sleep and wake states were determined by visual review of video monitoring and intracranial EEG data by an experienced epileptologist (M.H.). We acknowledge that detailed sleep staging from iEEG/video alone is not standardized; therefore, this analysis focused on binary wake–sleep differentiation, which is more robust from behavioral and EEG context cues. Formal interrater reliability testing was not performed, which represents a limitation of the present study.

The iEEG data were recorded using Neuvo amplifiers (Compumedics Neuroscan, Abbotsford, Victoria, Australia). The system uses a Sigma‐Delta converter with a high oversampling rate. Anti‐aliasing is realized by a first‐order analogue filter with a cut‐off frequency of 10 kHz. The decimation stage in the converter uses a digital anti‐aliasing filter with a SINC5 characteristic with a cut‐off frequency of 2035 Hz. To store the data finally at a sampling rate of 2 kHz, a second‐order Butterworth low‐pass filter with a cut‐off frequency of 800 Hz is used to prevent aliasing. It is a direct current amplifier without any hardware high‐pass filter.

**FIGURE 1:**
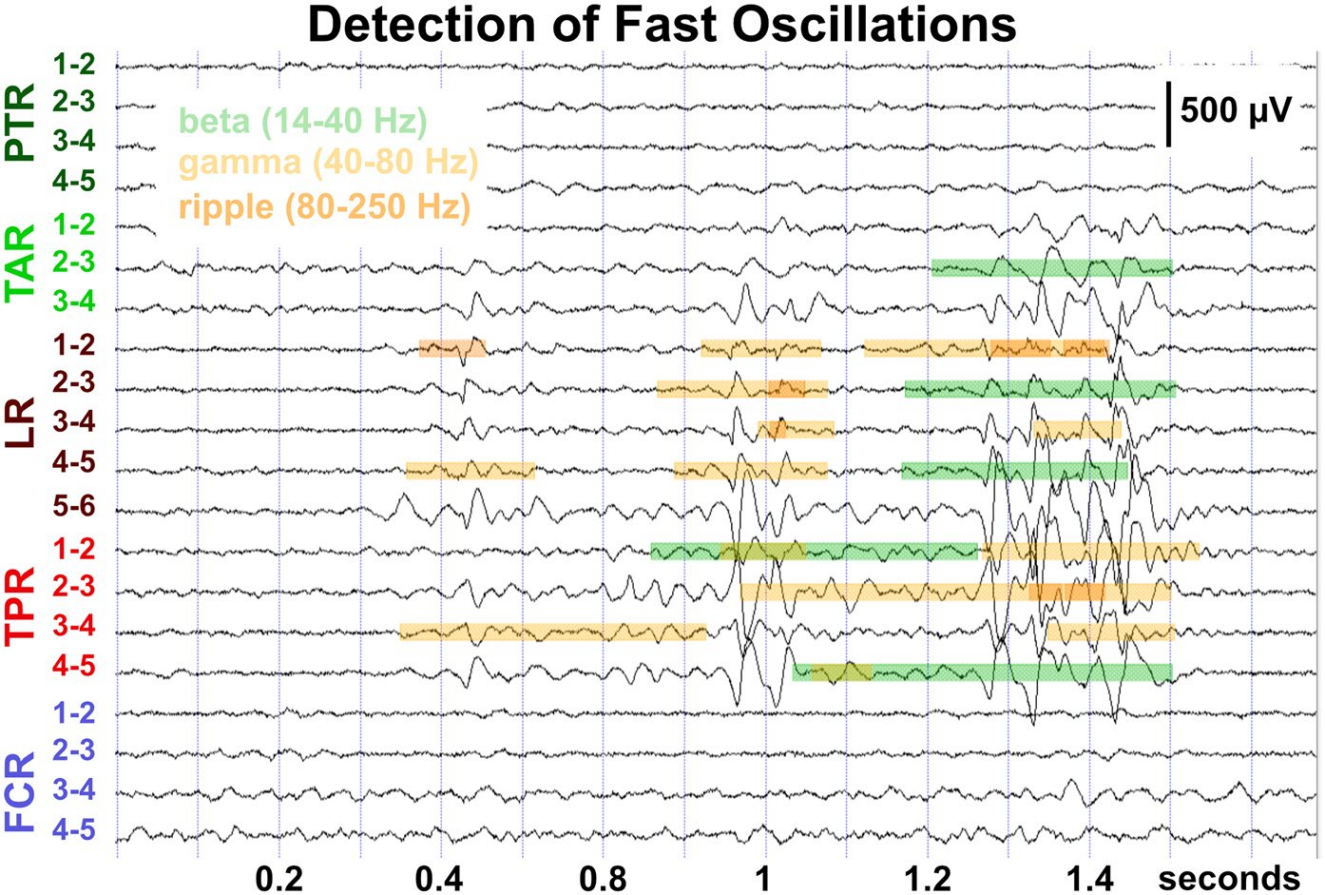
Detection of fast oscillations (FOs) using the toolbox Delphos^17^ within the AnyWave software.^33^ Signal traces of bipolar channels of five intracranial Stereo‐EEG electrodes shown (high‐pass filter: 1 Hz, notch filter: 50 Hz, no low‐pass filter). Markers for beta (14–40 Hz, green), gamma (40–80 Hz, yellow) oscillations, and ripples (80–250 Hz, orange) shown on marked EEG traces. Note the spatiotemporal overlap of different types of markers reflecting the co‐occurrence of FOs in different frequency bands of intracranial EEG recordings. Illustration of P12 (Table 1), same patient as Figure 2.

All recordings had a sampling rate of 2 kHz except for patient ID 11, where only iEEG data with a sampling rate of 1 kHz were available. The iEEG data were visually reviewed in multiple montages with AnyWave software,^33^ using a high‐pass filter with a cutoff frequency of 0.3 Hz, without low‐pass filtering. EEG channels with muscle artifacts due to an inverse breach rhythm^34^ or technical artifacts were removed from the analysis. Within AnyWave, the Delphos toolbox^17^, ^35^ with standard settings was used to detect FOs in the beta (12–40 Hz), gamma (40–80 Hz), and ripple (80–250 Hz) bands and epileptic spikes (spikes) using a bipolar montage (Figure 1). Detector performance was verified through visual inspection of detected events across representative recordings using the Delphos viewer tool, which includes whitening and time‐frequency visualization functions. This quality‐control step ensured that the automated detection was operating appropriately on the dataset.

### 2.5 Classification of iEEG contacts

Based on the available clinical data, iEEG contacts were classified into different groups. iEEG contacts which were part during the first second of the onset of clinical seizures were classified as belonging to the seizure onset zone (SOZ). The SOZ contacts always contained the contacts of the structural lesion as it was identified on MRI. All iEEG contacts that recorded interictal epileptiform discharges were classified as belonging to the irritative zone (IZ). The IZ was determined visually based on clinical EEG reports and confirmed through direct visual inspection of the iEEG recordings by the authors. In rare cases where SOZ channels were not part of the clinical IZ definition, they were included in the IZ group for this evaluation.

To determine electrodes implanted in eloquent brain areas, the results of clinical electrical stimulation were used. In patients without electrical stimulation, electrodes within the eloquent cortex were identified using the Automatic Anatomical Labeling atlas.^30^ Only electrodes located in eloquent cortex regions (primary sensory‐motor cortex, the primary language or primary visual cortex) with a probability of >70% according to the atlas were included in the ELOQ group.

Contacts outside the mesial temporal lobe, the IZ, the SOZ, and the ELOQ group were classified as OTHER, resulting in a group of neocortical electrode contacts to adjust for widespread neocortical sleep‐related changes in FO rates. Contacts within the ELOQ group were excluded from the normative group OTHER as their task‐related oscillations reflect functional processing specific to eloquent regions and differ markedly from background activity in non‐eloquent cortex.^36^, ^37^, ^38^ As we evaluated bipolar iEEG signals in bipolar montages, contact pairs were classified as SOZ or IZ if either contact belonged to one of these zones. If there was an overlap between the ELOQ group and the SOZ or IZ, these contacts were classified as SOZ or IZ contacts.

### 2.6 Wake and sleep effect on FO rates in different frequencies

To identify FO rates that were specific for the epileptic focus (SOZ or IZ), we subtracted the median FO rates of electrode contact pairs in the group OTHER from the rates of electrode contact pairs within the epileptic focus. For each patient, we computed the median rate across all channels within each group (SOZ, IZ, or OTHER), which accounts for the variable number of channels per region while remaining robust to outliers. We used subtraction rather than ratio‐based normalization to preserve interpretable rate units (events/minute) rather than dimensionless ratios, which facilitates direct comparison of absolute FO rates across different vigilance states (wake vs. sleep). This approach yields focus‐specific FO rates representing activity above the regional background level.

To account for variance across the OTHER contacts and provide a complementary validation of our subtraction approach, we additionally applied robust z‐scoring normalization. For each patient and frequency band, we computed z‐scores for FO rates in the epileptic focus (SOZ and IZ) using the median calculated during one brain state and median absolute deviation of FO rates measured from the OTHER contacts across sleep and wake. This z‐scoring approach standardizes the focus‐specific FO rates while accounting for the variability in background activity across different electrode placements.

**FIGURE 2:**
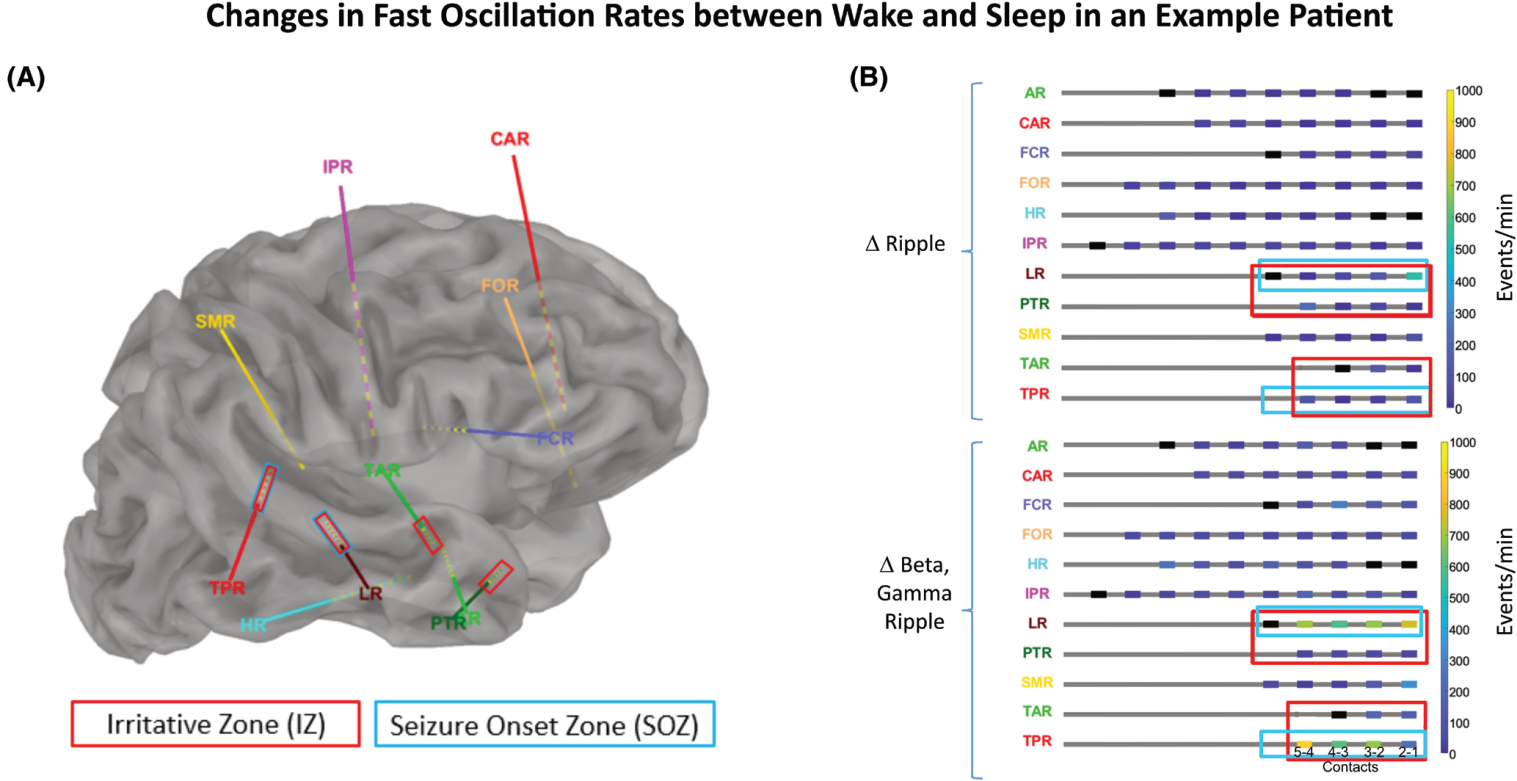
Detection of fast oscillations in an example patient with a focal cortical dysplasia type II in the right posterior superior temporal gyrus. (A) Implantation scheme with nine invasive Stereo‐EEG (iEEG) electrodes implanted in the right frontal, central, parietal, insular and temporal cortex. The irritative zone (IZ) contacts are marked with red rectangles and the seizure onset zone (SOZ) contacts are marked with blue rectangles. (B) Rate increase of ripples (upper part) and of broadband BGR (lower part) for each iEEG electrode from wake to sleep iEEG recordings. Please note the more pronounced detection rate changes when considering rate changes in the beta, gamma, and ripple frequency band together (lower panel) instead of focusing on the ripple frequency band alone (upper panel). Illustration of P12 (Table 1), same patient as Figure 1.

**FIGURE 3:**
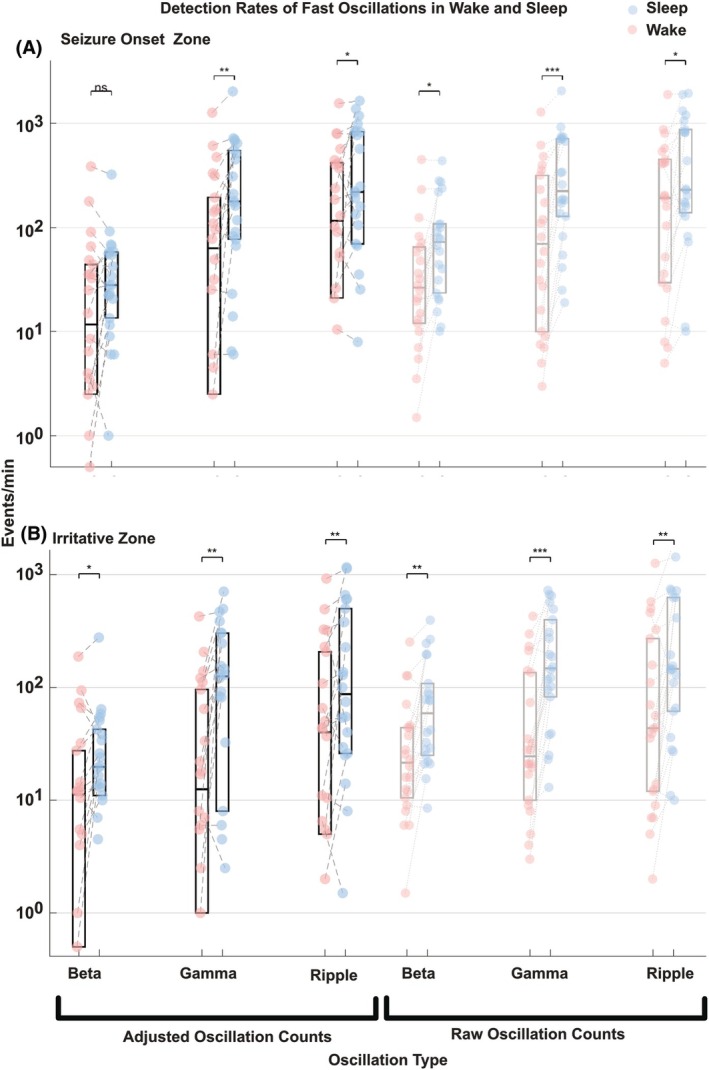
Comparison of detection rates between wake and sleep for beta and gamma oscillations and ripples across all patients. (A) Seizure onset zone (SOZ). (B) Irritative zone (IZ). Raw detection rates within the SOZ or IZ are illustrated as dots connected with gray lines between wake (pale red) and sleep (pale blue; right side of each panel). Detection rates adjusted by subtracting the fast oscillations (FO) rates measured in the OTHER electrodes group (details in methods: Classification of iEEG contacts) are illustrated in pink (wake) and blue (sleep, left side of each panel). Boxplots for raw detections are shown in gray and boxplots for adjusted detections in black. Significance level: *p < 0.05, ** *p* < 0.01, *** *p* < 0.001, ns: not significant. Note that for the SOZ we see a substantial increase for the adjusted gamma oscillations and ripples between wake and sleep (FDR‐corrected Wilcoxon signed‐rank test), but not for the beta frequency band. For the IZ, we see a substantial increase for the adjusted beta and gamma oscillations and ripples (FDR‐corrected Wilcoxon signed‐rank test). Note that the gray and black boxes of ripples in SOZ are largely overlapping with no correction effect, which is likely because ripples are highly specific to the SOZ.

The FO rates were evaluated for the SOZ and IZ for each frequency band (beta, gamma, and ripple) separately for wake and sleep states (example patient, Figure 2). To evaluate the effect of the brain states during wake and sleep on FOs in each frequency band, we tested whether FO rates increased from wake to sleep, separately for each frequency band, using non‐parametric Wilcoxon signed‐rank tests for median FO rates across all patients (Figure 3).

### 2.7 Statistical analysis

We used a nonparametric Wilcoxon signed‐rank test with FDR correction to account for multiple comparisons across the three frequency bands beta, gamma, and ripple frequency bands. This testing was applied to test whether each band showed changes in FO rate at the population level for all patients (Figure 3).

**FIGURE 4:**
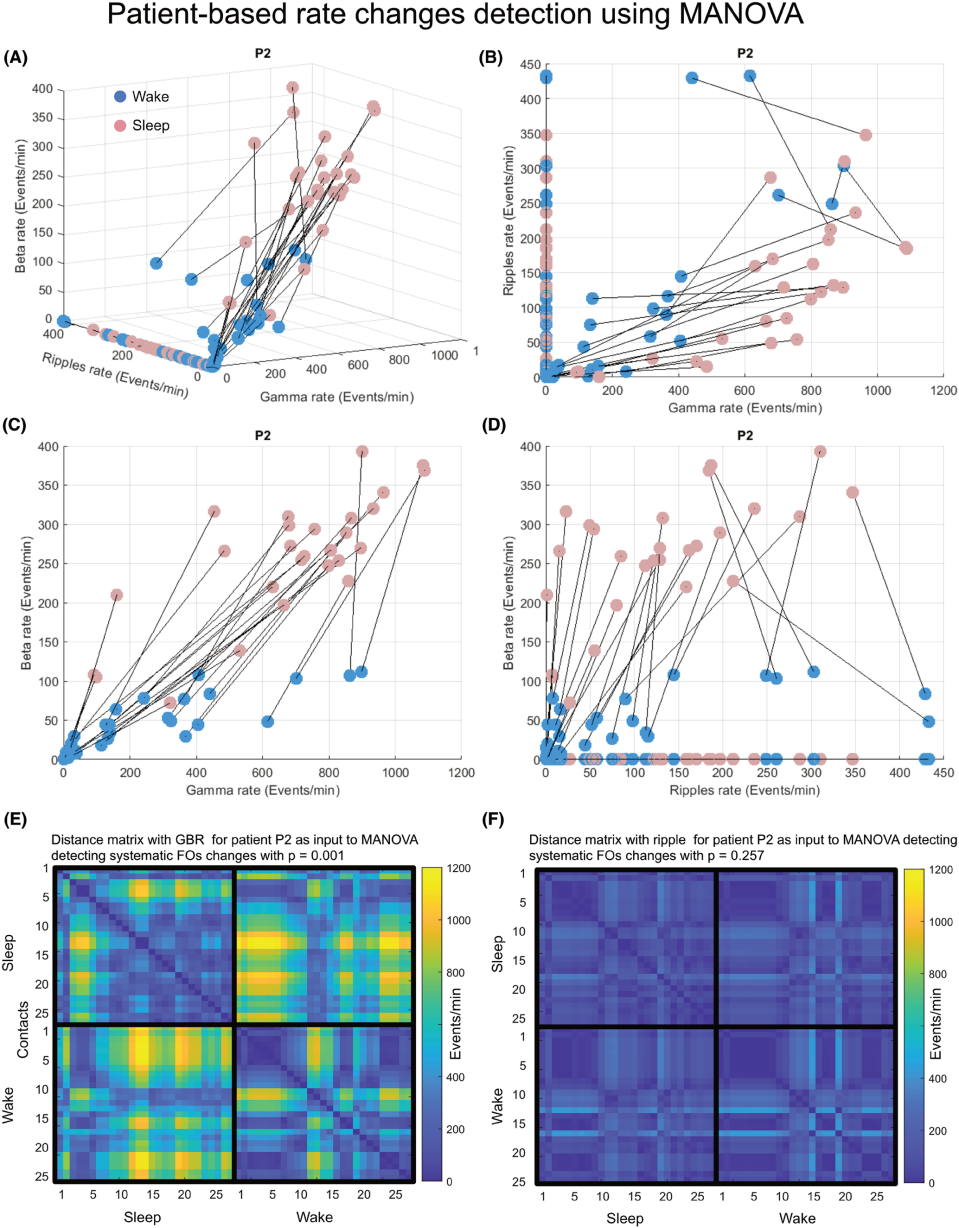
Changes in fast oscillation rates between wake and sleep across IZ contacts analyzed by MANOVA in patient P2. (A) Representation of fast oscillation rates during wake (pink) and sleep (blue) in 3D space along beta, gamma, and ripple axes. To compare the effectiveness of BGR versus ripple analysis alone, we replotted the same fast oscillation rate data points using only the ripple axis. Note that using all BGR provides larger separation between sleep and wake states. (B) 2D presentation of the 3D space presented in A with gamma rates on the *x*‐axis and ripples on the *y*‐axis. (C, D) The same as B with different 2D perspectives. (E) Distance matrix map of BGR fast oscillation rate differences measured between IZ contacts during the same states (upper left and lower right squares), or across wake and sleep states (lower left square). (F) The same as in E but for ripples alone. Note that using BGR enables identification of differences between wake and sleep that were not distinguishable in this patient with ripple analysis alone.

For the patient‐by‐patient analysis, we employed distance‐based Multivariate Analysis of Variance (MANOVA).^39^ This method offers the following advantages: 1: MANOVA calculates the distance of changes in FO rates while accounting for the spatial organization of detection rates through a Euclidean distance matrix across all contacts. 2: Simultaneous multiband analysis. The method allows for the inclusion of FO rates from all bands ‐ beta, gamma, and ripple (BGR) ‐ simultaneously. Each band's FO rate is represented on an axis within a 3D feature space, creating one data point for wake and one for sleep (Figure 4A–D). The Euclidean distance between the sleep and wake states in this 3D space is then calculated to perform MANOVA. We applied MANOVA to analyze FO rates for all bands combined (BGR) as well as for each band individually, ensuring a fair comparison between the combined BGR and the individual bands (Figure 4). Using MANOVA analysis allows processing the same data sample size independently of the number of bands included in the analysis. For a single band, the data are represented in a 1D space, whereas for BGR, the data are represented in a 3D space. In all cases, the sample size is determined by the number of contacts, which remains constant for a given patient. This approach provides a robust framework for analyzing FO rate changes across wake and sleep states, capturing the multidimensional complexity of BGR dynamics. While analyzing more frequency bands typically improves our ability to distinguish between sleep and wake states, this advantage diminishes when different bands show conflicting patterns of change.

MANOVA applies a nonparametric permutation‐based statistical approach to test whether the *F* value measured from the real data is larger than the ones produced by randomly shuffled data across the groups. The rows of multivariate observations were permuted by randomly exchanging their position; thus, sleep and wake labels were exchanged under the assumption of the null hypothesis—that no true difference exists between the groups. If the real *F* value is higher than 95% of the shuffled *F* values, then the effect is considered to be significant for a given patient.

We compared the outcome of MANOVA analysis regarding the detections of FO changes across wake and sleep comparisons for each frequency band (beta, gamma, ripple, and for all together BGR) in the patient's group perspectives: those with versus without sleep‐related epilepsy, and those with frontal versus extra‐frontal epilepsy.

We used a linear mixed‐effects model (LMM) to assess how sleep–wake state varies by region (SOZ, IZ, and OTHER) and event type (beta, gamma, ripple, and spikes), including region and event type as fixed factors along with their interaction. We modeled patient identity as a random intercept to account for inter‐individual variability and the repeated‐measures structure of the data. This modeling framework allows us to simultaneously evaluate multiple categorical predictors and their interactions on a continuous dependent variable, while appropriately modeling correlated measurements within patients. The LMM approach effectively mirrors the logic of a MANOVA, in that it examines the multivariate influence of multiple categorical factors, but it provides the crucial advantages of mixed modeling, namely robustness to unbalanced data and varying trial counts per patient, and the incorporation of random effects that capture subject‐level variability. Thus, the LMM yields a flexible, statistically rigorous multilevel analog of a MANOVA that is suited to our clinical dataset.

We used Chi‐square tests to examine three clinical hypotheses, using a significance level of α < 0.05. with Bonferroni correction for multiple comparison. We tested as well for associations between significant FO rate changes in:

1. Patients with versus without sleep‐related epilepsy.
2. Frontal versus non‐frontal lobe epilepsy.
3. FCD type II versus other histopathology (excluding three patients without histopathological FCD subtype classification).

## 3 RESULTS

### 3.1 iEEG contact distribution

We analyzed iEEG contacts across all patients, categorized into three groups: the irritative zone (IZ, 29 ± 16 contacts), the seizure onset zone (SOZ, 14 ± 12 contacts), and OTHER regions (30 ± 21 contacts).

### 3.2 General FO rate changes between wake and sleep

At the group level, the raw FO rates in the beta, gamma, and ripple bands within the SOZ and the IZ showed a significant increase during sleep as compared with wake (*p* < 0.05; Wilcoxon signed‐rank test with FDR correction; Cliff's delta for beta, gamma, and ripple in SOZ of 0.38, 0.45, and 0.28 and in IZ of 0.44, 0.59, and 0.38, respectively). Moreover, in the group OTHER, FO rates in the beta and gamma (*p* < 0.01 and *p* < 0.001; Wilcoxon signed‐rank test with FDR correction; Cliff's delta of 0.32 and 0.59, respectively) bands showed significant change but not ripples. Therefore, these findings within the SOZ or IZ may reflect a global sleep‐induced increase in FO rates across all brain regions, rather than a phenomenon specific to SOZ or IZ. To test whether SOZ and IZ regions exhibit an increase in FO rates that is beyond a general effect, we adjusted SOZ and IZ rates by subtracting the rates measured from contacts of the group OTHER (Median raw values for groups, IZ, SOZ, and OTHER see Appendix S1). Because unadjusted values were not necessarily specific for the epileptic focus, they were not further evaluated, and we continued with the adjusted values.

### 3.3 FO rates change in SOZ and IZ are beyond a general wake and sleep effect

At the group level and after adjustment for the general effect of sleep (details in Section 2.6), FOs in the SOZ still showed substantially higher rates during sleep as compared with wake for gamma oscillations and ripples (*p* < 0.01 and *p* < 0.05; Wilcoxon signed‐rank test with FDR correction; Cliff's delta of 0.40 and 0.28, respectively). However, the effect seen before adjustment in beta oscillations did not survive after adjustment by the group OTHER (Figure 3A). FO rates in the IZ substantially increased from wake to sleep recordings for all three bands (*p* < 0.05, *p* < 0.01, *p* < 0.01; Wilcoxon signed‐rank test with FDR correction, Cliff's delta of 0.30, 0.42, and 0.32 for beta, gamma, and ripple, respectively, Figure 3B). In contrast, no significant change was observed for the group OTHER for any band proving that our adjustment eliminated any general sleep effects. These results demonstrate that the effects we report in SOZ and IZ are beyond any general effect that might be seen in other regions. Interestingly, a closer look at Figure 3A shows that the adjustment did not change the rates of ripples much in the SOZ in contrast with other bands that were affected by the adjustment procedure. Our choice of subtracting the rates measured from contacts of the group OTHERS was driven by keeping our approach simple and interpretable. Yet, this approach did not account for the variance across OTHERS. Thus, we applied a robust z‐scoring based on the OTHERS contacts' activity (see Section 2.6 for more details). This procedure produced the same significant results shown by the subtraction approach, namely, significant increases in gamma and ripple for SOZ and in beta, gamma, and ripples in IZ (*p* < 0.05; Wilcoxon signed‐rank test with FDR correction; see Figure S1). This normalization did not show any sleep–wake effect in OTHERS contacts, similar to the subtraction approach.

### 3.4 Dissociation between FOs and epileptic spikes

To address concerns that our FO findings might be driven by spike contamination or detector artifacts, we independently studied spike detections. While spike rates might increase during sleep, spike and FO rate changes were largely dissociated. Specifically, concurrent sleep–wake changes in both spikes and FOs occurred within the SOZ in only 46% of the patients and within the IZ in 37% of the patients, where significant FO changes were observed. In contrast, 54% and 63% of patients showed significant sleep–wake FO rate changes without corresponding spike rate changes in SOZ and IZ, respectively, indicating that FO dynamics are not simply driven by spike activity.

### 3.5 Linear mixed‐effects model confirms regional and event‐type specificity

In a complementary analysis, we applied a linear mixed‐effects model to test the influence of each FO band and spikes on sleep–wake differentiation, considering band type and region as fixed effects (including their interaction) and patient variability as a random effect. Table 2 summarizes the estimated marginal means (EMMs) for Region (SOZ, IZ, and OTHERS). SOZ regions showed the highest estimated mean (emmean = 107.92, SE = 24.79, 95% CI [59.34, 156.51]), indicating robust sleep–wake differentiation. IZ regions exhibited an intermediate estimated mean (emmean = 77.87, SE = 24.04, 95% CI [30.75, 124.98]), while the iEEG channels of the group OTHERS showed the lowest estimated mean (emmean = 18.59, SE = 23.87, 95% CI [−28.18, 65.37]), reflecting minimal or uncertain effects.

**TABLE 2:** Estimated marginal means for sleep–wake state across cortical regions from the linear mixed‐effects model.

Region	Emmean	SE	df	Asymp.LCL	Asymp.UCL
SOZ	107.92	24.79	Inf	59.34	156.51
IZ	77.87	24.04	Inf	30.75	124.98
Other	18.59	23.87	Inf	−28.18	65.37

*Note*: The table reports the estimated marginal means (emmean), standard errors (SE), and asymptotic 95% confidence intervals lower and upper (asymp.LCL–asymp.UCL) for each region included as a fixed factor in the linear mixed‐effects model. Patient identity was modeled as a random effect to account for repeated measures. SOZ regions show the highest estimated sleep–wake values, followed by IZ regions (IZ without SOZ), whereas other cortical sites exhibit the lowest and least certain estimates, as indicated by wide confidence intervals spanning zero.

Critically, when examining event types (Table 3), spikes showed the weakest sleep–wake differentiation effect (emmean = 11.85, SE = 24.84), while ripples (emmean = 156.29) and gamma oscillations (emmean = 148.70) showed the strongest effects. This pattern confirms that our findings reflect genuine FO dynamics rather than spike‐related artifacts, as spikes and FOs demonstrate independent sleep–wake modulation profiles.

**TABLE 3:** Summary of estimated effects for each event type in the mixed‐effects analysis.

Event	Emmean	SE	df	Asymp.LCL	Asymp.UCL
Beta	33.07	24.84	Inf	−15.61	81.75
Gamma	148.69	24.84	Inf	100.02	197.38
Ripple	156.29	24.84	Inf	107.61	204.97
Spike	11.85	24.84	Inf	−36.83	60.53

*Note*: The table reports the estimated marginal means (emmean), standard errors (SE), and asymptotic 95% confidence intervals lower and upper (asymp.LCL–asymp.UCL) for each event category (Ripple, Gamma, Beta, Spike) entered as levels of the categorical predictor in the model. Ripple and Gamma events exhibit the highest estimated means, whereas Beta and Spike events show lower and less certain estimates, reflecting substantial variability across subjects.

### 3.6 Patient‐based rate changes of beta, gamma, ripples, and BGR

To apply a comprehensive analysis at the patient‐by‐patient level, we used MANOVA, which provides a robust framework for analyzing FO rate changes across wake and sleep states, capturing the multidimensional complexity of BGR dynamics. This analysis considered the spatial organization of FO changes in feature space and allows simultaneous multiband analysis, ensuring a fair comparison between the combined BGR and the individual bands (see Section 2 for more details).

**FIGURE 5:**
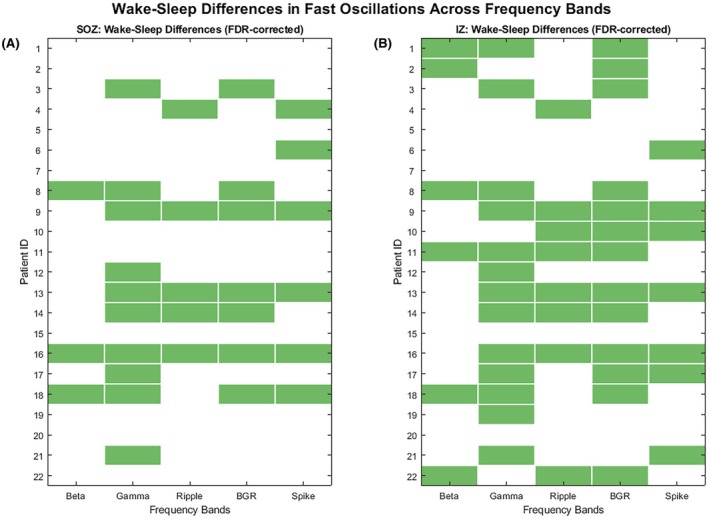
Patient‐level statistical significance patterns of wake–sleep fast oscillation rate differences in seizure onset zone (SOZ) and irritative zone (IZ). Green cells indicate statistically significant differences (*p* < 0.05, MANOVA, FDR‐corrected) between wake and sleep states for each frequency band and individual patient (ID 1–22). White cells represent non‐significant differences (FDR‐corrected *p* ≥ 0.05). (A) SOZ recording showing significant wake–sleep differences most frequently in gamma (10/22 patients, 45%) frequency band, followed by BGR (7/22 patients, 32%), then ripple (5/22 patients, 23%), and beta (3/22 patients, 14%). Spikes rate showed significant wake–sleep differences in (6/22 patients, 27%). (B) IZ recordings demonstrating significant differences most commonly in gamma (13/22 patients, 59%) and broadband BGR (13/22 patients, 59%) frequency bands, followed by ripple (8/22 patients, 36%) and beta (6/22 patients, 27%). Spikes rate showed significant wake–sleep differences in (7/22 patients, 32%). The superior performance of gamma and BGR likely reflects the multi‐band nature of wake–sleep oscillation changes in epileptogenic tissue, with this multi‐band activity appearing to focus in the gamma frequency band. Note that spikes rate behavior does not predict any frequency band behavior. Note that FDR correction was applied row‐wise per patient to control for multiple comparisons across frequency bands. Frequency band definitions: beta (14–40 Hz), gamma (40–80 Hz), ripple (80–250 Hz), broadband BGR (14–250 Hz).

Using the MANOVA approach within the SOZ, significant wake–sleep differences were most pronounced in the gamma band (10 patients), followed by BGR (7 patients), while beta and ripple bands showed more limited detection with three and five patients, respectively (Figure 5). The binary significance patterns reveal substantial patient‐to‐patient variability, with some patients (ID 3, 8, 9, 13, 14, 16, 18) showing significant differences across multiple frequency bands, while others showed no significant wake–sleep differences in any band.

Within the IZ, gamma oscillations showed the most consistent wake–sleep differences (13 patients), closely matched by BGR broadband changes (13 patients), reflecting that changes typically occur across multiple frequency bands simultaneously (Figure 5). In contrast, ripple and beta oscillations showed somewhat lower detection rates with 8 and 6 patients, respectively. Overall, gamma consistently showed the highest detection rates across both epileptogenic regions, while BGR provided comparable performance in the IZ. We applied MANOVA analysis to spike rates and found significant sleep–wake changes in 6 patients (27%) within the SOZ and in 7 patients (32%) within the IZ. Importantly, we did not observe consistent sleep–wake differentiation patterns across the different FO bands, nor between FOs and spikes. These results reinforce the central message of our manuscript: a comprehensive assessment of all relevant signals—including FOs across different frequency ranges and spikes—is essential for capturing the diversity and complexity of epileptogenic networks in FCD patients.

### 3.7 FOs in patients with sleep‐related epilepsy, frontal lobe epilepsy, and FCD type II

A comparison between patients with and without sleep‐related epilepsy (defined as two‐thirds of seizures occurring during sleep in presurgical video‐EEG monitoring^6^) revealed an association between sleep‐related epilepsy and increased rates of gamma oscillations (*p* < 0.05, Chi‐square test, Bonferroni‐corrected for four frequency bands: beta, gamma, ripple, broadband BGR). However, there was just a trend toward increased broadband BGR (*p* = 0.07, Chi‐square test, uncorrected) within the SOZ. No relevant associations were found for other frequency bands within the SOZ nor for any frequency bands within the IZ (Appendix S1). There was also no relevant association between sleep‐related FO‐rate changes for frontal versus non‐frontal SOZ or FCD type II versus other histopathological types (Chi‐square test, details Appendix S1). No difference in sleep‐related epilepsy was observed between patients with frontal and non‐frontal epilepsy (*p* = 0.8, Chi‐square test, Appendix S1).

## 4 DISCUSSION

### 4.1 Key information in gamma band ideally supplemented by broadband BGR

Our analysis shows a systematic increase in FO rates from wake to sleep, specific to the epileptic focus, in iEEG recordings of patients with FCD. In contrast to our initial hypothesis within the SOZ, not broadband (BGR) rates but gamma oscillations showed the most substantial and consistent increase of FOs. The increase in rates of gamma oscillations within the SOZ was significant in the subgroup of patients with proven sleep‐related epilepsy. Broadband oscillations (BGR) showed the second most frequent changes at the patient level. Within the SOZ, significant changes in oscillation rates in the beta and ripple frequency bands were less common. Often, significant changes occurred simultaneously across multiple frequency bands, with gamma showing the highest concordance with other bands. Only in single patients did significant changes in beta and ripple bands occur when gamma band and broadband analyses remained nonsignificant. So, analyzing rate increases in the gamma band supplemented by broadband analysis appear to be most promising to detect the FO rate increases in the SOZ.

Within the IZ, the increase in FO rates from wake to sleep was more pronounced than in the SOZ, with both gamma and broadband (BGR) frequencies showing equally strong and highly concordant changes. Beta and ripple frequency bands less frequently showed relevant changes.

These findings highlight that focusing on the gamma frequency band alone supplemented by broadband frequencies yields the most distinct separation of FO rates between wake and sleep. Beta and ripple frequency bands, while less effective for this distinction, often align with gamma and broadband BGR findings and provide additional relevant information within both SOZ and IZ in a few patients.

Gamma‐band oscillations co‐occurring with interictal epileptiform discharges in SOZ are known as good markers for the SOZ.^40^, ^41^, ^42^ Similarly, it is known that increased rates of ripples are specific markers for the SOZ.^43^, ^44^ In our study, the specificity of ripples for the SOZ is reflected by the fact that they did not change much after adjustment by the group OTHER, as they were rare outside the SOZ (Figure 3). However, we did not aim to evaluate the accuracy of those parameters in detecting the SOZ, but to characterize their increase from wake to sleep, which might help unravel relevant differences of the SOZ and IZ between these brain states. The prominence of gamma oscillations during sleep compared with wake within the SOZ might reflect more synchronized neuronal activity in the core epileptogenic zone,^45^ whereas the equal importance of BGR in the IZ could indicate more asynchronous broadband activity in the surrounding tissue. This aligns with our group's previous observation of high‐amplitude spectral peaks in the SOZ.^14^

A broad range of frequencies is known to be spatially related to the IZ and SOZ in patients with FCD. Beta and gamma oscillations contribute valuable information about the epileptic focus in FCD patients.^19^ Gamma oscillations spatially overlap with dysplastic neurons in FCD type II, which establishes a very close pathophysiological relation.^46^ Ripples are known to reflect disease severity in FCD patients.^47^ Moreover, it has been demonstrated that gamma oscillations are not only increased within the SOZ but also in directly adjacent epileptogenic tissue,^20^ which might align with our finding that the inclusion of the irritative zone (IZ) improves detection of FO rate increases from wake to sleep. All these insights together reinforce the notion that detection rates of gamma oscillations supplemented with broadband BGR analysis could be relevant to capturing the complexity of the IZ and SOZ and the modulation of FOs between wake and sleep iEEG recordings of FCD patients.

### 4.2 Sleep‐related changes and potential mechanisms in FCD

Neuronal excitability in focal epilepsy undergoes significant changes during sleep. Studies using single‐pulse electrical stimulation in patients with focal epilepsy show higher amplitude of early N1 responses during sleep, suggesting increased excitability followed by intense inhibition compared with wakefulness.^48^ In FCD patients, our observation of increased FO rates during sleep might reflect such heightened excitability, particularly as typical FCD discharge patterns (rhythmic spike, polyspike wave complexes at 1–3 Hz with fast discharges) not only become more frequent but also spread to surrounding non‐lesional areas during NREM sleep.^49^ Paradoxically, while increased excitability occurs during sleep, rates of interictal epileptiform discharges often decrease during sleep in patients with FCD.^7^, ^15^

The complex relationship between sleep and epileptic activity potentially stems from FCD's integration within the thalamocortical network. Dysplastic neurons exhibit connectivity patterns resulting in stronger phase‐amplitude coupling in regions with greater dysplastic neuron density.^46^ This integration may explain our finding of significantly increased gamma oscillations specifically in patients with proven sleep‐related epilepsy, with similar trends observed in broadband BGR. However, these broadband differences between wake and sleep are not unique to FCD patients, as similar patterns quantified through spectral slope changes have been observed in other types of focal epilepsy, suggesting increased excitation during sleep may be a broader phenomenon.^50^ Whether the frequency‐specific patterns we observed—particularly the prominence of gamma oscillations in the SOZ—are unique to FCD or generalizable to other focal epilepsies remains to be determined and warrants investigation in broader patient cohorts. Contrary to previous reports,^51^ in our cohort we found no evidence that frontal lobe FCD patients experience more frequent sleep‐related epilepsy compared with FCD patients with SOZ outside the frontal lobe. Whether sleep‐related seizures are associated with lesion location or intrinsic properties of the dysplastic tissue remains undetermined, and the exact mechanisms by which sleep influences FCD epileptogenicity require further investigation.^15^

### 4.3 Semiautomated detection of FOs

FO detection is crucial for epilepsy diagnostics and identifying the epileptic focus. Using semi‐automated detection tools, like the Delphos toolbox,^35^ we processed large datasets efficiently, allowing for consistent evaluation of FOs across beta, gamma, and ripple bands. The semi‐automated detection enabled fast and standardized analyses of 1‐h sleep and wake iEEG recordings, identifying frequency‐specific changes that visual review might have missed. While this method reduces bias and speeds up analysis, it is not without limitations, such as susceptibility to filter artifacts and noise. To minimize errors, each recording was visually screened for artifacts, but the risk of including false positive detections remains, highlighting the need for further refinement of automated methods. Yet, such false positive detections are unlikely to be seen during one brain state but not another, which minimizes the bias of automated detection on our results.

Combining FOs from different frequency bands can be more informative than focusing on single frequency bands but is limited by the reviewer's capacity to interpret large amounts of data. With the development and ongoing improvements of artificial intelligence, analyzing more complex information is becoming much easier and more available for clinical use, even for healthcare providers without coding experience.^52^

### 4.4 Limitations

In this study, we relied on semiautomated detections. The limitations of semiautomated detections are typically related to thresholding and might contain filter artifacts and technical artifacts.^53^ However, the implemented detector efficiently avoided these artifacts by analyzing the data in the wavelet domain, and it was shown to be specifically insensitive to mistakenly identifying epileptic spikes as FOs.^17^, ^35^ The risk of erroneous marking was further reduced by visual inspection of each dataset.

Although the differentiation between wake and sleep recordings was done as thoroughly as possible, we did not perform sleep staging considering standard sleep stages. The study also did not account for temporal changes in fast oscillation rates within the same sleep state or consider the potential effects of age or developmental stage on fast oscillations. Additionally, we did not differentiate between FCD type 2a and type 2b, and some patients included in the study did not have histopathological confirmation of FCD.

### 4.5 Future directions

In future studies, a more detailed differentiation of sleep stages and co‐occurrences with K‐complexes and sleep spindles will probably give even more insight.^54^ Additionally, the extended analysis of sleep and wake intervals over multiple days could provide more valuable data.^55^ Investigations of FO rate changes in relation to epileptic seizures, cross‐frequency coupling, or data‐driven analyses might also yield important findings.^56^

## AUTHOR CONTRIBUTIONS

MFK, NS, and MH contributed to the study design and conceptualization. NS collected all data and performed initial analysis steps under the supervision of MH. MFK conducted advanced statistical analysis and modeling. AB contributed to statistical analysis. MFK, NS, and MH contributed equally to data analysis and interpretation. MD provided expert advice on technical aspects of biosignal analysis. YLH, NR, AM, DMA, VSA, PCR, JMN, SD, TD, HU, and ASB contributed to results interpretation. NR advised on the implementation of the Delphos detector. TD and HU reviewed MRI findings. NS, MFK, and MH wrote the manuscript. MH supervised the project. AB, MD, YLH, NR, AM, DMA, VSA, PCR, JMN, SD, TD, HU, and ASB reviewed the manuscript and contributed to its final version. All authors read and approved the final manuscript.

## FUNDING INFORMATION

YLH (LI1904/2‐1) and MH (HE6844/3‐1; project ID for both: 468174690) were funded by the German Research Foundation (DFG).

## CONFLICT OF INTEREST STATEMENT

Reinacher, PC receives research support from Else‐Kroener‐Fresenius Foundation (Germany) and Fraunhofer Society (Germany), received honoraria for lectures from Arkana (Germany), and is a consultant for Boston Scientific (USA), Inomed (Germany), and Brainlab (Germany). Schulze‐Bonhage, A has received research support from BIAL, Precisis, and UNEEG, and personal honoraria for lectures or advice from Angelini Pharma, JAZZ Pharma, Precisis, UCB, and UNEEG. Urbach, H. received honoraria for lectures from Biogen, Eisai, Mbits, Lilly, Bayer, is supported by the German Federal Ministry of Education and Research, and is coeditor of Clin Neuroradiol. Heers, M has received support for conference participation from Jazz/GW Pharmaceuticals and Precisis, speaker's honoraria for lectures from Eisai and Arkana, and research funding from the German Research Foundation (DFG). N Schoon, MF Khazali, A Brandt, M Dümpelmann, Y Li Hegner, N Roehri, DM Altenmüller, V San Antonio‐Arce, JM Nakagawa, S Doostkam, T Demerath have no conflicts of interest. We confirm that we have read the Journal's position on issues involved in ethical publication and affirm that this report is consistent with those guidelines.

## Supporting information


Appendix S1:

## Data Availability

The datasets presented in this article are not readily available because of ethical restrictions. Requests to access the datasets should be directed to marcel.heers@uniklinik-freiburg.de.
