# Supplementary material for: Sleep enhances gamma oscillations in the seizure onset zone and broadband activity in the irritative zone of focal cortical dysplasia
Source: Epilepsia Open. 2026 Jan 20;11(2):435–51. doi: 10.1002/epi4.70215 (PMC13051985; doi:10.1002/epi4.70215)
Supplement: Supplementary file 1 — Appendix S1: [file EPI4-11-435-s001.pdf]

Suppl. 1

Median Detections per Frequency Band with Delphos Detector

**Seizure Onset Zone (Events per minute: median Q1, Q3)**					
Beta		Gamma		Ripple	
Wake	Sleep	Wake	Sleep	Wake	Sleep
27 (12, 63)	73 (25, 105)	70 (12, 295)	73 (25, 105)	192 (31, 422)	230 (144, 862)
**Irritative Zone (Events per minute, median Q1, Q3)**					
Beta		Gamma		Ripple	
Wake	Sleep	Wake	Sleep	Wake	Sleep
22 (11, 44)	59 (26, 90)	25(12, 120)	148 (86, 376)	44 (12, 243)	224 (130, 708)
**OTHER (Events per minute, median Q1, Q3)**					
Beta		Gamma		Ripple	
Wake	Sleep	Wake	Sleep	Wake	Sleep
12 (8, 28)	30 (11, 52)	10 (3, 14)	33 (20, 87)	9 (2, 70)	35 (10, 102)

Numbers of intracranial EEG contacts per patient within different regions

	mean	median	STD	range	total
SOZ	14	11	12	1-45	299
IZ	29	29	16	3-52	628
OTHER	30	24	21	5-87	667

Chi-Square Analyses:

Analysis 1: Sleep-related epilepsy: distribution in frontal vs Non-frontal locations

SRE in frontal locations: 8/13 (61.5%)

SRE in Non-frontal locations: 5/9 (55.6%)

Chi-square = 0.079, p = 0.779

Analysis 2: SRE vs oscillation patterns

**Seizure Onset Zone**	**Irritative Zone**
Beta:<br>SRE: 4/13 significant (30.8%)<br>Non-SRE: 1/9 significant (11.1%)<br>Chi-square = 1.170, p = 0.279	Beta:<br>SRE: 3/13 significant (23.1%)<br>Non-SRE: 4/9 significant (44.4%)<br>Chi-square = 1.119, p = 0.290
Gamma:<br>SRE: 9/13 significant (69.2%)<br>Non-SRE: 1/9 significant (11.1%)<br>Chi-square = 7.246, **p = 0.007****<br>**(p = 0.04* Bonferroni-corrected)**	Gamma:<br>SRE: 10/13 significant (76.9%)<br>Non-SRE: 4/9 significant (44.4%)<br>Chi-square = 2.424, p = 0.119
Ripple:<br>SRE: 4/13 significant (30.8%)<br>Non-SRE: 1/9 significant (11.1%)<br>Chi-square = 1.170, p = 0.279	Ripple:<br>SRE: 6/13 significant (46.2%)<br>Non-SRE: 2/9 significant (22.2%)<br>Chi-square = 1.316, p = 0.251
BGR:<br>SRE: 8/13 significant (61.5%)<br>Non-SRE: 2/9 significant (22.2%)<br>Chi-square = 3.316, p = 0.069	BGR:<br>SRE: 9/13 significant (69.2%)<br>Non-SRE: 5/9 significant (55.6%)<br>Chi-square = 0.430, p = 0.51

Analysis 3: Frontal vs Non-frontal oscillation patterns

**Seizure Onset Zone**	**Irritative Zone**
Beta:<br>Frontal: 2/13 significant (15.4%)<br>Non-frontal: 3/9 significant (33.3%)<br>Chi-square = 0.976, p = 0.323	Beta:<br>Frontal: 4/13 significant (30.8%)<br>Non-frontal: 3/9 significant (33.3%)<br>Chi-square = 0.016, p = 0.899
Gamma:<br>Frontal: 5/13 significant (38.5%)<br>Non-frontal: 5/9 significant (55.6%)<br>Chi-square = 0.627, p = 0.429	Gamma:<br>Frontal: 7/13 significant (53.8%)<br>Non-frontal: 7/9 significant (77.8%)<br>Chi-square = 1.316, p = 0.251
Ripple:<br>Frontal: 3/13 significant (23.1%)<br>Non-frontal: 2/9 significant (22.2%)<br>Chi-square = 0.002, p = 0.962	Ripple:<br>Frontal: 5/13 significant (38.5%)<br>Non-frontal: 3/9 significant (33.3%)<br>Chi-square = 0.060, p = 0.806
BGR:<br>Frontal: 5/13 significant (38.5%)<br>Non-frontal: 5/9 significant (55.6%)<br>Chi-square = 0.627, p = 0.429	BGR:<br>Frontal: 9/13 significant (69.2%)<br>Non-frontal: 5/9 significant (55.6%)<br>Chi-square = 0.430, p = 0.512

Analysis 4: FCD Type II vs other pathologies

Seizure Onset Zone	Irritative Zone
Beta:<br>FCD2: 3/12 significant (25.0%)<br>Other: 1/6 significant (16.7%)<br>Chi-square = 0.161, p = 0.688	Beta:<br>FCD2: 3/12 significant (25.0%)<br>Other: 3/6 significant (50.0%)<br>Chi-square = 1.125, p = 0.289
Gamma:<br>FCD2: 6/12 significant (50.0%)<br>Other: 3/6 significant (50.0%)<br>Chi-square = 0.000, p = 1.000	Gamma:<br>FCD2: 8/12 significant (66.7%)<br>Other: 4/6 significant (66.7%)<br>Chi-square = 0.000, p = 1.000
Ripple:<br>FCD2: 3/12 significant (25.0%)<br>Other: 2/6 significant (33.3%)<br>Chi-square = 0.138, p = 0.710	Ripple:<br>FCD2: 3/12 significant (25.0%)<br>Other: 3/6 significant (50.0%)<br>Chi-square = 1.125, p = 0.289
BGR:<br>FCD2: 5/12 significant (41.7%)<br>Other: 3/6 significant (50.0%)<br>Chi-square = 0.112, p = 0.737	BGR:<br>FCD2: 6/12 significant (50.0%)<br>Other: 5/6 significant (83.3%)<br>Chi-square = 1.870, p = 0.171

FCD: focal cortical dysplasia, SRE: sleep related epilepsy, BGR: beta-gamma-ripple

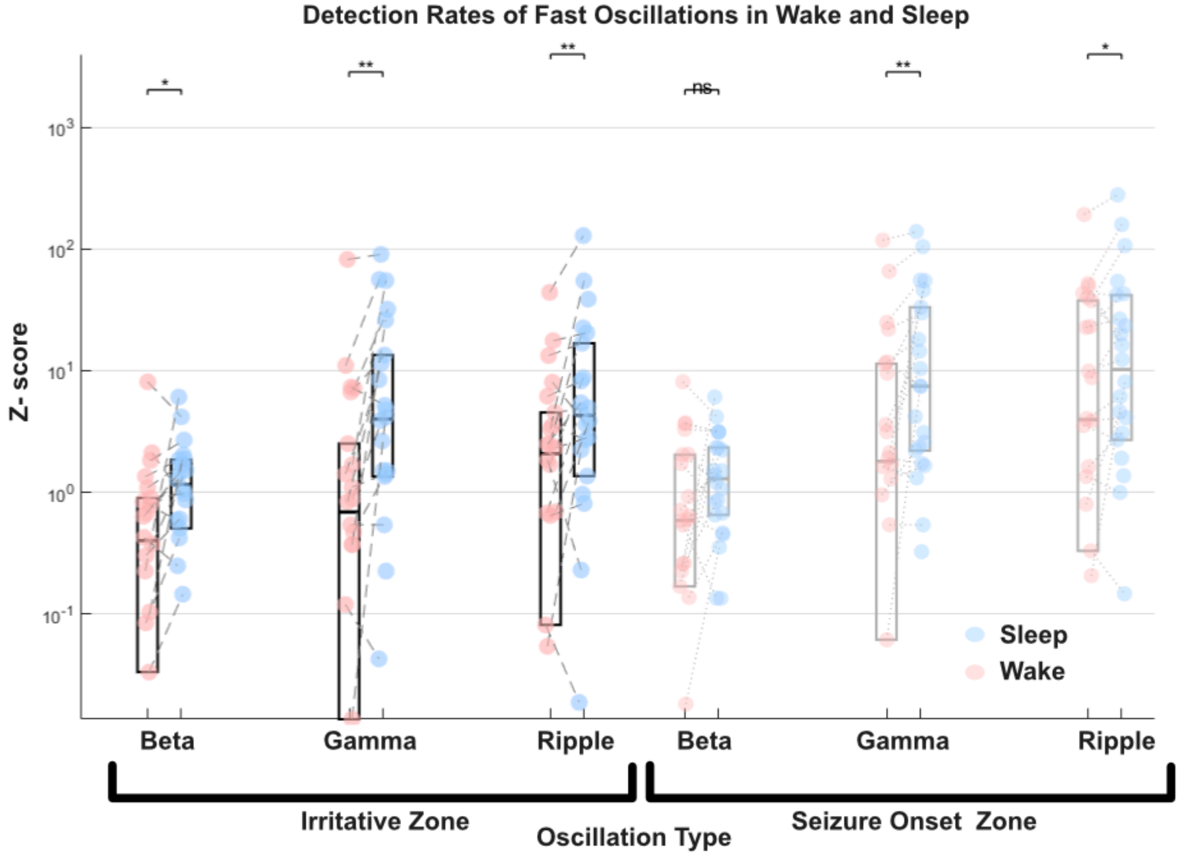

Suppl. Fig. 1. Z-score normalized fast oscillation rates confirm sleep-wake differences in epileptogenic regions. Detection rates of fast oscillations (FOs) in wake (pink) and sleep (blue) after z-score normalization based on OTHERS channel activity for each patient. Left: Irritative zone (IZ, black boxplots). Right: Seizure onset zone (SOZ, gray boxplots). Each dot represents one patient, with dashed lines connecting wake-sleep pairs for the same patient. The SOZ shows significant increases during sleep for gamma oscillations and ripples (**$p < 0.01$ and *$p < 0.05$, respectively; Wilcoxon signed-rank test with FDR correction), but not beta oscillations (ns). The IZ shows significant increases during sleep for all three frequency bands (beta, gamma, and ripples; *$p < 0.05$ or **$p < 0.01$). These results replicate the findings obtained with median-based subtraction (Figure 3), confirming that sleep-wake FO rate differences in epileptogenic regions are robust to the normalization method. Significance levels: **$p < 0.01$, *$p < 0.05$, ns: not significant.
